# 

**Published:** 1899-04

**Authors:** 


### PERSONALS.

Governor Stone, of Pennsylvania, sent to the Senate, which body confirmed, the reappointment of Drs. Harry Walters and W. Horace Hoskins to the Pennsylvania State Board of Veterinary Medical Examiners, in February.

At a theatre party in Philadelphia in February were to be noted Drs. W. L. Williams, John W. Adams, Leonard Pearson, and W. Horace Hoskins.

Dr. J. C. McKenzie, of Rochester, N. Y., after five years of earnest labor on the scientific board of the Horseshoers' National Protective Association, retires from active duty, much to the regret of all those who have watched and profited by his intelligent and well-directed labors in this direction.

The January meeting of the Kansas State Board of Agriculture was favored by several important papers by veterinarians—the subject of "Swine-plague and Black-leg Protective Inoculation," by Dr. Paul Fischer; the "Serum-treatment for Hog-cholera," by Dr. D. E. Salmon; "The Farmer His Own Veterinarian," by Dr. M. Stalker.

Drs. J. J. Fallon and W. F. Walsh, of New York City, are active members of the Master Horseshoers' organization of that city, and were actively interested in the ball given in November by that organization.

Dr. R. P. Lyman, of Hartford, Conn., fills the rôle of veterinarian to the Hartford County Milk Commission, which prepares modified milk for the use of children and invalids.

Dr. H. W. Eliot, formerly of Ansonia, Conn., is now a resident of Willets Point, N. Y. He has added an M.D. degree to his title of veterinarian.

Dr. H. B. Felton, of Philadelphia, has been on the sick list for many months, but is now convalescent, and hopes to resume practice again at an early day.

Dr. Leonard Pearson was a frequent visitor at Harrisburg during the sessions of the State Legislature.

Dr. A. J. Savage, of Colorado Springs, Colo., is just recovering from a fractured humerus, received by being thrown down on entering his carriage.

Dr. H. D. Fennimore, of Knoxville, Tenn., did not accept the appointment of meat-inspector to which he was assigned, at Kansas City, by the Bureau of Animal Industry in January.

Dr. J. M. Richardson, V.S., who graduated from the New York College of Veterinary Surgeons, in the class of '98, is attending the Royal College of Veterinary Surgeons, London, England.

Dr. Robert M. Mullings, a graduate of the class of '98, New York College of Veterinary Surgeons, is with the United States Army and stationed at Washington, acting as veterinarian. He has the rank of second lieutenant.

The Minneapolis dog-show in March had as their veterinarian Dr. Charles E. Cotton, of that city, a graduate of the Veterinary Department of the University of Pennsylvania.

Dr. Enoch H. Moore, of Philadelphia, has disposed of his practice to Dr. H. B. Cox, formerly of Merchantville, N. J. Dr. Moore has entered upon a mercantile venture.

Dr. Charles E. Cotton, of Minneapolis, Minn., has been re-elected veterinarian to the city board of health for two years.

Dr. M. J. Sexton, of Minneapolis, a graduate of the Ontario Veterinary College, class of '95, was selected in February as assistant inspector for that city in the dairy inspection service.

Dr. Thomas M. Waldron, of Greensburg, Pa., is much interested in the breeding and handling of trotters.

Dr. Leonard Pearson, State Veterinarian, addressed the State Dairymen's Association of Pennsylvania in March.

Dr. C. P. Lyman, of Boston, returned to active duties in the early part of March, after a month's leave of absence from his duties at the Veterinary Department of Harvard University.

Dr. N. Rectenwald, of Pittsburg, will attend the Congress of Veterinarians at Baden Baden, in August, and return in time for the meeting of the American Veterinary Medical Association in New York in September.

Dr. W. H. Fry, of Pine Grove Mills, Centre County, Pa., takes an active interest in local politics, and has filled official places in his district for more than twenty years.

Dr. Leonard Pearson, State Veterinarian, has resumed his lectures at the State College at Bellefonte, Pa.

Dr. J. C. Foelker, of Allentown, Pa., was elected a school director for his district in February. It is his first acceptance of a position, semi-political in character, but one he is well equipped to fill.

Dr. D. S. White, of Columbus, addressed the Ohio dairymen in February on "Prevention and Treatment of Injuries and Diseases of the Udder."

Dr. A. J. Schrieber, of Philadelphia, has been much interested in loaning assistance to one "Suzette," of the *Evening Bulletin*, who has been doing heroic service in relieving the distress of some of Philadelphia's poor during the severe winter.

Dr. James Beatty, of Philadelphia, who successfully passed the civil-service examination as an inspector and received an assignment for duty at Buffalo, was unable to accept the appointment.

Dr. E. Mayhew Michener, of North Wales, Pa., has recovered in great measure from the serious injuries received while pursuing his profession.

Dr. N. C. Lazarus, of Nazareth (more recently of Stroudsburg, Pa.), has become afflicted with almost total blindness, which has caused his relinquishment of active work in the profession.

Dr. A. L. Compton, of Perry, Mich., has sold his practice to J. E. Ward, of Williamstown. Dr. Compton has taken the practice of Dr. T. H. Bates.

Dr. T. H. Bates, of Williamstown, Mich., has removed to Port Huron, in the same State.

Dr. George R. White fills the rôle of city veterinarian of Nashville, Tenn.

Dr. H. L. Ramacciotti, of Omaha, Neb., was presented with a diploma and beautiful bronze medal by the officials of the Trans-Mississippi Exposition for his devoted efforts in promoting the success of the Exhibition.

Dr. R. P. Steddom, an inspector in the Bureau of Animal Industry, for some time connected with the Texas-fever quarantine service west of the Mississippi, has been selected for a special mission in the interests of the Bureau to investigate the live-stock industry of Puerto Rico.

Dr. John P. O'Leary, formerly stationed at East Buffalo, has been transferred to the Texas-fever quarantine service, with headquarters at Kansas City.

